# Factors contributing to tourism demand at major Japanese hot springs

**DOI:** 10.1371/journal.pone.0274681

**Published:** 2022-09-15

**Authors:** Nagi Medai, Naoyuki Okamoto, Yu Ogasawara, Katsuya Hihara

**Affiliations:** 1 Department of Tourism Science, Tokyo Metropolitan University, Hachiouji-City, Tokyo, Japan; 2 Former Department of Tourism Science, Tokyo Metropolitan University, Hachiouji-City, Tokyo, Japan; 3 Graduate School of Public Policy, The University of Tokyo, Bunkyo-ku, Tokyo, Japan; Universiti Malaysia Sabah, MALAYSIA

## Abstract

Hot springs are a major tourism resource in nature-based tourism, and the hot springs market is one of the biggest sectors in wellness tourism markets. In the present study, we examine factors contributing to tourism demand for major hot spring resorts in Japan using ordinary least squares regression models and generalized linear mixed models, and compare the estimation results. The results show that significant factors in most of our models are quality of accommodations and the degree of dependence on inbound demand. Furthermore, the number of non-Japanese languages supported on websites of hot spring resorts has a significant impact on inbound demand. Since the results of the present study cover more than 80 hot spring sites, the results highlight common important factors for hot spring resorts. Such widely applicable factors have been missing in previous studies, and the present study fills this research gap.

## Introduction

Wellness tourism is a form of tourism that is currently getting more and more popular around the world. Since wellness is associated with maintaining health rather than curing specific diseases, wellness tourism is defined as a form of tourism that attempts to preserve and promote the health of tourists [1, 2]. Wellness facilities and programs associated with wellness tourism have become global tourism attractions since the end of the 20th century [2]. Because of the expansion of the wellness tourism market, wellness tourism has already been studied extensively.

The market scale of wellness tourism was estimated to be $4.2 trillion in 2017, and it has been reported that the market is also associated with 10 major related markets, including spas and wellness-related real estate [3]. One of the major sectors in related markets is thermal/mineral springs. The thermal/mineral springs sector is defined as “revenue-earning business establishments associated with the wellness, recreational, and therapeutic uses of waters with special properties” [3]. The thermal/mineral spring sector, which is based on an on-site source of nature offering spa services and related services has become a major component of wellness tourism [2]. The market scale of this sector is approximately $56.2 billion, which is comparatively small as compared to $639.4 billion, which was the largest market size in 2017. However, the thermal/mineral spring sector maintains a similar growth rate to the other sectors. This market is mainly concentrated in the Asia-Pacific region and Europe, with 95% of the total revenue of the sector coming from these areas. Specifically, Europe has a market of $21.7 billion, and the Asia-Pacific region has a market of $31.6 billion. Thus, the Asia-Pacific region has big presence in this sector. Detailed statistics are provided by the Global Wellness Institute (2018) [3]. Although the Global Wellness Institute (2018) distinguishes the thermal/mineral spring sector from wellness tourism, this sector is treated as one type of spa activity in health and wellness tourism in tourism research [3].

The leading countries in terms of revenue of thermal/mineral springs in the Asia-Pacific region are China and Japan, due to the existence of thousands of hot springs in Japan and huge investments in hot spring resorts in China [4]. Mineral springs in Japan are referred to as *onsen* with the first *onsen* dating back to AD 737 [2, 5]. According to a report by the Ministry of the Environment of Japan (2021), there were 2,971 hot spring resorts with accommodation facilities in Japan in 2020 [6]. Lee and King (2010) reported that hot springs in Japan are highly competitive, as compared to those in Taiwan [7], and Kamata et al. (2010) evaluated their attractiveness [8]. In addition, there are few data analyses dealing with international tourists in Japan [9]. Most of the studies on thermal/mineral springs were conducted in Europe, China, and Taiwan. These studies were mainly questionnaire-based and evaluated the attributes of visiting customers for a few hot spring resorts, the quality of customer service provided by the resorts, or the strategies with regard to spa facilities in the resorts [7–9]. However, few econometric approaches have examined the impacts of various factors on the demand for hot spring resorts using statistical data, despite the large market size of the thermal/mineral spring sector in wellness tourism.

The present study estimates factors affecting the demand for hot spring resorts in Japan using an econometric approach. We use an ordinary least squares (OLS) model and a generalized linear mixed model (GLMM) with independent variables, such as the quality of accommodations, the number of natural and cultural resources, and accessibility, all of which are considered to be important factors for the attractiveness of hot spring resorts in tourism studies. This analysis reveals the important factors related to the demand for hot springs based on economic data, which is in contrast with questionnaire data that are dominant in tourism studies dealing with hot springs. The demand for a hot spring resort might be considered to be the number of people staying in the city where the hot spring resort is located. However, the people staying in the city are not necessarily users of the hot spring. Therefore, the present study uses not only the number of people staying in a city, but also the bath tax paid by hot springs users as demand data. We compare the analytical results for the number of guests in the city and for the bath tax in the city as the dependent variable. In addition, in order to analyze factors affecting inbound demand on hot spring resorts in Japan, we analyze a model with the number of inbound overnight guests as a dependent variable. The number of non-Japanese languages on a tourism association website and the number of duty-free stores are used as independent variables in the model.

An outline of the present study is as follows. We present a literature review in the second section, in which we clarify our research position by explaining previous studies into hot spring resorts both in Japan and elsewhere. The data and regression models used in the present study are described in the third section. In the fourth section, we show the results obtained by analyzing the data using the models described in the third section. We discuss the results in the fifth section, and finally summarize the present study and its results in the sixth section.

## Related literature

Many studies have focused on hot spring tourism in China and Taiwan. Deng (2007) proposed an improved method of importance-performance analysis and applied it to questionnaire survey data for overseas tourists in a hot spring resort in Taiwan [10]. He reported that “ease of obtaining staff attention and help” is the most important tourist satisfaction attribute. Hsu et al. (2009) used a Bayesian network mechanism with a linear structural relation model to investigate the impact of customer service on tourist loyalty by using survey data for a hot spring resort in Taiwan [11]. Their results showed that customer service, website function, and local attributes have an impact on tourist loyalty, and that local attributes are the most important among these three considerations. Lee et al. (2009) conducted a questionnaire survey for domestic visitors in Taiwan to estimate factors determining the attractiveness of hot spring resorts [12]. They found that gender, age, and perception for importance of accommodations significantly affected the frequency of visits.

Studies are focusing on the intention to revisit a hot spring, which is an important theoretical factor as well as a valuable factor for the business practice. Lin (2013) investigated factors affecting the intentions of Taiwanese tourists to revisit hot spring resorts. The results show that destination characteristics, cuisine experience, and psychological well-being were important factors [13]. In addition, Lin (2014) proposed a structural model including the effects of cuisine experience and psychological well-being on the intention to revisit Taiwan to investigate the relationship with self-health perception [14].

Customer service offered by facilities in hot springs is also an important factor to evaluate the quality of hot springs resorts. Chen et al. (2013a) and Chen et al. (2013b) focused on customer service [15, 16], and Chen et al. (2013a) estimated the elements of customer service by an analytic hierarchy process (AHP) for hotel visitors in hot spring resorts in Hong Kong [15]. They showed that the most important weighting factor for customer service is personal service for both younger and older customers, followed by environment attributes, such as location and cleanliness of bathrooms. Chen et al. (2013b) additionally conducted a questionnaire survey for older customers aged over 50 in major hot spring regions in Taiwan to examine the reliability and validity of customer service by factor analysis, and they performed customer segmentation by cluster analysis [16]. Liu et al. (2019a) used a partial least squares structural equation to model service quality and proposed indices to measure service quality at hot spring resorts [17]. They collected data from a hot spring resort in southern China and showed that there were six components of service quality: water quality, customer service, facilities, surroundings, alternative activities, and convenience. Liu et al. (2019b) additionally investigated the impact of the on-site experiences of travelers on loyalty through questionnaire results obtained from visitors to the resort in southern China [18].

Mi et al. (2019) used Web data, rather than questionnaires, to identify factors to improve customer satisfaction regarding hot spring tourism [19]. In their study, comments regarding hot spring resorts in China obtained from online services Ctrip and Qunar were analyzed using the grounded theory approach and interpretive structural modeling. They demonstrated that environmental quality, special resources, convenience, food, service quality, and facilities are the factors affecting customer satisfaction.

Competitiveness of destination is another focus. Lee and King (2006) showed that the competitiveness of hot spring tourism in Taiwan depended on the tourism destination resources and attractors, tourism destination environments, and tourism destination strategies, using the Delphi method [20]. Lee and King (2009) suggested the importance of safety and security at hot spring resorts using survey items, which extended the questionnaires of Lee and King (2006), and the Delphi method [21]. Based on these studies, Lee and King (2010) evaluated the potential value of hot spring resorts in Taiwan and in Japan by the AHP method [7]. The results showed that among the factors determining the competitiveness of hot spring resorts, tourism destination strategies have the highest weight. By comparing the competitiveness of Japanese and Taiwanese hot spring destinations, they found that Japanese hot spring destinations are more competitive.

As studies of hot spring resorts outside of China, Clark-Kennedy and Cohen (2017) found that the key motivators for going to hot springs in Australia were indulgence, relaxation, escape, connection with nature, and taking time out [22]. Medina-Muñoz and Medina-Muñoz (2013) conducted a questionnaire survey of European wellness tourism visitors to Gran Canaria [23]. They presented an exploratory study of what tourists consider to be important and the attributes of tourists in Gran Canaria.

Several papers studied about Japan. Kamata et al. (2010) estimated the attractiveness of hot spring regions in Japan by conjoint analysis, which covers 20 hot spring regions [8]. They set quality, amusement (comfort, restaurants, events, *ryokan* size, etc.), and atmosphere, as well as travel time and cost as factors that determine attractiveness. The above study showed that the attractiveness of hot spring regions generally decreases as the time distance from Tokyo or Osaka increases. Using factor analysis, Kamata and Misui (2015) found that there are seven factors that motivate people to travel to hot spring resorts in Japan, and that these people can be segmented into five types [24]. They argued that soothing is an important common factor for these non-homogeneous tourists. Kurata and Ohe (2020) analyzed the factors affecting the prices of accommodations near Dogo Onsen, which is a major hot spring resort in Japan, using a spatial error model [25]. They examined the effect of being a *ryokan* and the proximity of accommodation facilities to each other on price. Choi et al. (2018) use factor analysis and cluster analysis to categorize the attributes of Korean tourists who used *ryokan* in Japan and their decisions about staying in *ryokan* [26]. Shapoval et al. (2018) found that Japanese food and shopping influence the intention of inbound tourists to visit Japan, although the survey was not specifically designed for hot spring tourists [9].

Most tourism studies dealing with hot spring resorts have focused on service quality, customer service, and customer satisfaction. The studies conducted in Japan have investigated the effect of being a *ryokan*, Japanese original accommodation style, and the location of accommodations. However, these studies are based on questionnaire data obtained from a limited number of hot spring regions. Although Mi et al. (2019) and Kurata and Ohe (2020) used Web data, the number of studies using Web data or public statistical data is limited, and they did not conduct an analysis of city-level data [19, 25].

In addition, COVID-19 has been reported since early 2020 according to the WHO [27], and global tourism markets have been greatly impacted [28]. This has changed individual behavior and social customs, which include attitudes to social distancing and trust of government or destination in travel decisions [29]. Japanese tourism markets, including hot springs markets, were affected by the pandemic, and Handler and Kawaminami (2022) reported that a large population is less concerned about visiting hot springs during the pandemic [30]. It is not clear yet how individual attitudes and behavior for visiting hot springs are going to change during and after the pandemic, specifically getting back to or being completely different from the situation in 2019 because, according to the WHO, we are still in the pandemic as of the end of June 2022 [27]. Statistical analyses of COVID-19 influence obtained in [29, 30] are based on survey data. It would be very important to accurately gauge the impact on demand factors under the still fluid pandemic circumstances, if such studies are available that use open data that can be made available in a time-consistent manner across a wide range of hot springs, and use methods that can be validated before and after the pandemic.

Policy suggestions or general insights for tourism industries at hot springs in general could be desirable based on studies of broad, stable, and consistent open data in a wide range of facilities, but collecting such data is not necessarily easy as the scarcity of such papers has been indicated in previous studies, which are mostly based on survey data for particular areas. Therefore, studies actually based on these data across a wide range would be very important.

To examine what factors robustly influence demand at various hot spring resorts, using public statistics data might be efficient. However, some public data are not usually provided for hot spring resorts. In this context, social Web data, for instance from Tripadvisor or Expedia, were used to determine customer satisfaction [31, 32] from the viewpoint of hospitality industries. While there are limited number of studies using Web data for analyzing hot spring resorts [19, 25], Web data, specifically rating data for hotels, could be used to capture the quality of hotels that influence customer satisfaction in the market.

In addition, for hot springs, robust studies that make use of a statistical method specific to counting data and that incorporate the idiosyncratic random effects of hot spring destinations, such as the GLMM (gamma or Poisson distribution with a normal distribution), have not conducted to our knowledge.

The present study applied regression analysis (OLS and GLMM) to combined public source, governmental, and Web data, at the city/municipality level of more than 80 hot spring resorts in Japan in order to examine whether these factors that have been shown to be effective for determining customer satisfaction and the attractiveness of hot springs in previous studies using survey data or covering a limited number of hot springs in Japan indeed have significant effects on accommodation demand at the city/municipality level. By conducting these analyses, the present study clarifies relevant factors that could directly contribute to demand in hot spring regions. Furthermore, we also examine factors contributing to the demand for overnight stays by inbound guests, the results of which can help understand the demand of inbound tourists for hot spring resorts and can contribute to a comparative study of demand factors before and after the start of the pandemic. Thus, our results also reinforce the baseline before the pandemic if the transitional change in factors affecting hot spring demand from pre-pandemic to post-pandemic periods is examined.

## Data and models

### Data

It has been reported that there are 2,000 to 3,000 hot spring sites with accommodation facilities in Japan [6], and considering all of these sites is difficult due to their large number. Therefore, in the present study, we deal with only major hot springs. Major hot springs in the present study are defined as the 100 hot springs based on the “Hot Spring Destinations Best 100 by Number of Guests in 2010” [33], and we analyze the data for the cities and municipalities in which these hot spring resorts are located. Table 1 shows the ten hot spring resorts with the highest revenue according to the bath tax at the city/municipality level.

**Table 1:** Top 10 hot spring destinations ranked by number of the bath tax revenue in 2010 (Source: Nihon Onsen Kyoukai [33]).

Rank	Name	Prefecture	City	Bath taxes (JPY)
1	**Hakone-Onsen-Kyo**	Kanagawa	Hakone	683,722,000
2	**Atami-Onsen-Kyo**	Shizuoka	Atami	439,575,000
3	**Jyozankei**	Hokkaido	Sapporo	408,322,000
4	**Kinugawa & Kawaji/ Nikko-Yumoto & Chuzenji**	Tochigi	Nikko	384,695,000
5	**Ito**	Shizuoka	Ito	353,199,000
6	**Beppu**	Oita	Beppu	320,640,000
7	**Arima**	Hyogo	Kobe	277,250,000
8	**Okuhida-Onsen-Kyo/Takayama**	Gifu	Takayama	245,935,000
9	**Yamashiro/Yamanaka/Katayamazu**	Ishikawa	Kaga	233,133,000
10	**Yunokawa**	Hokkaido	Hakodate	203,679,000

We first use 1) the number of overnight guests as a dependent variable to explain the demand for hot spring sites. The number of overnight guests is obtained from Japan Voyage Navigator, which is a data platform service for local governments in Japan and is offered by the Japan Travel and Tourism Association, a public interest corporation.

Note that out of the number of guests in a municipality, we cannot distinguish between visitors who actually stay in hot spring accommodations in the municipality and visitors who stay in a nearby urban area but do not use hot spring accommodations, because we analyze not data for hot spring usage, but rather data for tourists who stay in a municipality that includes a hot spring. For example, the nationally famous Dogo hot spring resort is located in the city of Matsuyama, the prefectural capital of Ehime Prefecture, in Japan. Hence, there are many business hotels in the city. As a result, the number of overnight guests consists of the number of tourists staying at Dogo Onsen (hot spring accommodations) and the number of business tourists staying in the city center [25]. In the present study, we tackle this problem by including 2) bath tax revenue in our data set. The bath tax is a special-purpose tax imposed on bathing in mineral bath facilities. The standard tax rate is 150 yen per person per day, and all bathing guests at hot springs need to pay the tax. We treat this bath tax as an indicator of the number of users who stay at a hot spring site in a municipality, that is, the demand for the hot spring. Note that this indicator is affected by local people (not tourists) who use a hot spring as well as day-trip users of hot springs. In the case of Kurokawa hot spring, it has been reported that the number of uses of hot spring tickets, which are coupons for bathing, and the number of overnight-stay guests both transitioned in approximately the same manner. Moreover, there was no major difference between the number of overnight-stay guests at a hot spring resort and the number of bath tax revenues [34]. In the present study, the bath tax revenue data for each municipality are obtained from settlement statistics for each municipality, which are provided by the Ministry of Internal Affairs and Communications. In this regard, since Kuwana and Kushiro cities have higher bath tax rates than the standard rate, we exclude these two cities in our analysis when using models with bath tax revenue.

In the present study, the independent variables are the number and quality of accommodation facilities, the number of tourism resources, accessibility from major cities, and the quality of hot spring water, which are reported in existing hot spring studies to have an impact on the potential value and attractiveness of hot springs. Rátz (2008) reported that some hot springs in Japan have a high hurdle for inbound tourists to use their facilities due to language barriers and local transportation problems [5]. In addition, Deng (2007) pointed out that customer satisfaction in hot springs is affected by the ease of obtaining attention and help from staff [10]. Therefore, to our independent variables, we added the percentage of 3) inbound guests among total guests as an indicator of the degree of effort and success of a hot spring region in attracting inbound customers. For the number of accommodation facilities, we used 4) the number of *ryokan* and hotels by municipality from the data of the Economic Census Survey for Business Activity in 2016 collected by the Ministry of Internal Affairs and Communications [35]. As the quality of accommodations, we use 5) the percentage of *ryokan* and hotels with a rating of 4 or higher on Tripadvisor to the total number of *ryokan* and hotels. Tripadvisor is one of the most popular social media platforms worldwide in the tourism industry [31]. Although the platform maintains moderated online reviews, which biases the data, many studies use Web data in Tripadvisor to analyze customer satisfaction or service quality because the data contains an enormous amount of information [36]. For the number of tourism resources, we use 6) the number of natural resources by municipality and 7) the number of humanities resources excluding hot springs, which were obtained from the Tourism Resources Directory offered by the Japan Travel Bureau Foundation. For accessibility, we set the shortest travel time to each hot spring site as the travel time from 8) Tokyo Station, 9) Shin-Osaka Station, or 10) the nearest bullet train station in cities with a population of over a million. The quality of the hot spring water was taken from 11) the list of National Health Onsen Resorts published by the Ministry of the Environment and is expressed as a dummy variable. In addition, the present study uses 12) the number of non-Japanese languages offered on the website of a hot spring provided by the tourism association of its municipality and 13) the number of duty-free stores in the municipality as independent variables that might affect the demand for inbound guests at a hot spring resort. There are 85 municipalities for which data for all of these variables were available, and the present study analyzes these 85 municipalities.

Table 2 shows the data sources of the variables used in the present study and the basic statistics of the data. Note that the ‘year’ for some variables in Table 2 is not 2018. This is because surveys for *ryokan* and hotels and those for natural and cultural resources are not conducted every year. The present study assumes that the number of *ryokan* and hotels and the number of natural and cultural resources by municipalities have not significantly changed, as compared to 2016.

**Table 2:** Variables used in the present study and their sources.

Variables	Mean	SD	Year	Source
**1) Overnight guests**	1,723,219.79	1,484,328.13	2018	Japan Voyage Navigator (Japan Travel and Tourism Association) https://kankouyohou.com/en/
**2) Bath taxes (JPY)**	124,428,294.12	105,952,015.55	2018	Local financial situation survey (Ministry of Public Management, Home Affairs, Posts and Telecommunications) https://www.soumu.go.jp/english/index.html
**3) Inbound guests**	168,958.62	281,507.55	2018	Japan Voyage Navigator (Japan Travel and Tourism Association) https://kankouyohou.com/en/
**4) Ryokan and hotels**	82.74	63.09	2016	Economic Census Survey for Business Activity (Ministry of Internal Affairs and Communications) https://www.stat.go.jp/english/data/e-census.html
**5) Percentage of highly rated ryokan and hotels**	29.85	12.85	2018	Tripadvisor https://www.tripadvisor.jp/
**6) Natural resources**	1.56	2.21	2017	Tourism Resources Directory (Japan Travel Bureau Foundation) https://www.jtb.or.jp/page-search-tourism-resource/ (in Japanese)
**7) Cultural resources**	2.31	3.33	2017	Tourism Resources Directory (Japan Travel Bureau Foundation) https://www.jtb.or.jp/page-search-tourism-resource/ (in Japanese)
**8) Shortest time from Tokyo Station (minutes)**	165.67	54.44	2018	Route Information (Yahoo!Japan) https://transit.yahoo.co.jp (in Japanese)
**9) Shortest time from Shin-Osaka Station (minutes)**	193.59	65.73	2018	Route Information (Yahoo!Japan) https://transit.yahoo.co.jp/ (in Japanese)
**10) Shortest time from station in big city where the Shinkansen stops (minutes)**	115.13	54.67	2018	Route Information (Yahoo!Japan) https://transit.yahoo.co.jp/ (in Japanese)
**11) Dummy of the National Health Onsen Resorts**	0.22	0.42	2018	List of the National Health Onsen Resorts (Ministry of the Environment of Japan) https://www.env.go.jp/nature/onsen/area/ (in Japanese)
**12) Non-Japanese languages supported by tourism association websites**	2.84	2.03	2018	85 websites of the tourism association of each municipality (all are publicly accessible)
**13) Duty-free stores**	11.86	20.34	2018	Japan Voyage Navigator (Japan Travel and Tourism Association) https://kankouyohou.com/en/

All of the data in the present study were collected and analyzed in accordance with the terms and conditions of each data source indicated in Table 2. Collection of all data requires no special technology. Most of the data are publicly and readily available from the indicated sources. However, making use of some of the data sources requires payment (overnight guests or inbound guests from Japan Voyage Navigator). Other data are restricted to data for the time in which access is attempted without possibility to freely trace the data for a certain year (percentage of highly rated ryokan and hotels from Tripadvisor, three types of data for shortest time from Shinkansen stops, and non-Japanese languages supported by tourism association from tourism association websites of 85 municipalities). Therefore, such data cannot be accessed freely or publicly traced back to the data for the year in Table 2.

To maintain the necessary level of data sharing and replicability, we set up a minimum data set repository as part of the present study, excluding third-party data to which payment or legal permission is necessary to obtain access. Such excluded data are overnight guests or inbound guests from Japan Voyage Navigator. Anyone who obtains access to the restricted source of Japan Voyage Navigator according to the terms indicated on the website can add such data to our data repository and can completely reconstruct our data set.

### Models

We use an OLS regression model and a GLMM to estimate the effect of the variables shown in Table 2 as independent variables for the dependent variables. In order to compare the GLMM with the OLS using the Akaike information criterion (AIC), two models were set up for the GLMM: one assuming a gamma distribution for the error structure and the other assuming a Poisson distribution considering that the dependent variables are non-negative count data. Hence, for the GLMM, the error term is assumed to be either gamma or Poisson distributed. We use a log link function for the GLMM model, which is given by the following equation:

$$\ln\;E\left(y_{i}\right)=\beta_{0}+\sum_{j=1}^{p}\beta_{j}x_{ij}+r_{i},$$

i∈{1,2,…,n},

j∈{1,2,…,p},

$$r_{i}∼N(0,\sigma^{2})$$

where *i* and *j* are indices for municipalities and independent variables, respectively. Here, y_i_ denotes a dependent variable for municipality *i*, *β*_0_ is an intercept, *x*_*ij*_ is an independent variable *j* for municipality *i*, *β*_*j*_ is a parameter corresponding to the independent variable, and *r*_*i*_ is a random effect variable that is assumed to follow *N*(0, *σ*^2^).

In the present study, we consider three dependent variables, i.e., the amount of revenue from the bath tax, the number of overnight guests, and the number of inbound overnight guests in each municipality, in order to examine two types of demand: overall demand and inbound demand for Japanese hot springs. These variables are clearly affected by the number of accommodation facilities in each municipality. In the OLS models, the number of *ryokan* and hotels is included as an independent variable to adjust for this effect, and in the GLMM models, this number is set as an offset variable. The models with the bath tax revenue and the number of overnight guests as dependent variables are denoted as Model BT and Model OG, respectively, and the model with the number of inbound overnight guests as a dependent variable is denoted as Model FG. In addition, the OLS and GLMM regression models are denoted as O and G, respectively. For the distribution that the errors follow in the GLMM, P and G indicate Poisson distribution and gamma distribution, respectively. In the present study, the models to be tested are represented by a combination of these notations. For example, an OLS model with the bath tax revenue as a dependent variable is written as “Model BT-O”, whereas a GLMM model with the number of inbound overnight guests as a dependent variable and assuming a Poisson distribution for an error structure is written as “Model FG-G (P)”.

The independent variables for Model BT and Model OG are from variables in Table 2. Specifically, the independent variables are the percentage of highly rated *ryokan* and hotels; the number of natural resources and cultural resources; the shortest travel time to Tokyo Station, Shin-Osaka Station, or the nearest bullet train station in cities with a population of over a million from each municipality where a hot spring resort is located; a dummy variable indicating whether the hot spring resort is on the list of National Health Onsen Resorts; and the percentage of inbound guests among total overnight stay guests. For independent variables in Model FG, the percentage of inbound guests among the total number of overnight guests was excluded from Model BT and Model OG, whereas the number of non-Japanese languages supported by the tourist association website and the number of duty-free stores in each municipality were added. The software used for the computation was R version 3.6.2, and the standard lm function was used to estimate the OLS model. Moreover, lme4 version 1.1.26, was used to estimate the GLMM.

## Results

The computational results are shown in Table 3. We find that the statistically significant variables in both the OLS model and the GLMM are not very different. The AIC and the root-mean-square error (RMSE) are used to evaluate the models. Note that we cannot compare models with a continuous error structure and a discrete error structure. For all dependent variables, the AIC is lower for the GLMM with a gamma distribution for the error structure, than for the OLS model. It is also confirmed that the GLMM with a gamma distribution and the GLMM with a Poisson distribution produce a better fit than the OLS model in terms of the RMSE. On the other hand, the difference in the RMSE between GLMM (gamma) and GLMM (Poisson) was not very large.

**Table 3:** Estimation results for the ordinary least squares (OLS) model and the generalized linear mixed model (GLMM).

	Model BT						Model OG						Model FG						
	OLS		GLMM (Gamma)		GLMM (Poisson)		OLS		GLMM (Gamma)		GLMM (Poisson)		OLS			GLMM (Gamma)		GLMM (Poisson)	
	Model BT-O		Model BT-G (G)		Model BT-G (P)		Model OG-O		Model OG-G (G)		Model OG-G (P)		Model FG-O			Model FG-G (G)		Model FG-G (P)	
	Coef. (×10^-3)		Coef. (×10^3)		Coef. (×10^-3)		Coef. (×10^-3)		Coef. (×10^3)		Coef. (×10^-3)		Coef. (×10^-3)			Coef. (×10^3)		Coef. (×10^-3)	
**Intercept**	30965.43		13276.65	***	13359.90	***	947.98		9209.49	***	9136.47	***	-189.67			4881.00	***	4818.05	***
**Percentage of highly rated ryokan and hotels**	1912.75	**	18.02	**	18.28	**	21.28	*	15.33	**	15.73	**	5.04	*		31.50	**	31.01	*
**Natural resources**	4251.21		-23.83		-24.04		95.40		5.38		5.64		3.18			88.25		92.38	
**Cultural resources**	8428.88	**	44.58		42.80		72.77	*	43.52		42.25		6.55			18.33		20.12	
**Shortest travel time from Tokyo Station**	-451.22	*	0.37		0.35		-8.04	**	-0.06		-0.46		-0.35			4.24		4.24	
**Shortest travel time from Shin-Osaka Station**	226.86		3.11	*	3.01	*	-0.81		1.43		1.33		0.08			-1.51		-1.06	
**Shortest travel time from station in big city**	-396.66	*	-3.53	*	-3.36		-1.55		-2.40		-1.82		-0.15			0.02		-0.10	
**Dummy of the National Health Onsen Resorts**	71.43		-499.31	**	-498.6	**	-270.40		-490.43	**	-481.54	**	-92.32			-856.71	*	-862.32	*
**Percentage of inbound guests**	4305.18	***	29.75	**	31.26	**	65.70	***	40.67	***	39.26	***							
**Non-Japanese languages supported by tourism association websites**													39.41	**		176.19	*	179.01	*
**Duty-free stores**													1.25			10.21		10.37	
**Ryokan and hotels**	642.80	***					12.56	***					1.72	***					
**Standard deviation of Random effects**			639.94		639.69				660.54		659.60					1384.32		1384.05	
**Sample size**	83		83		83		85		85		85		85			85		85	
**AIC**	3256.96		1717.41		3234.17		2592.47		994.59		2574.71		2357.57			572.91		2189.76	
**RMSE**	28590348.89		0.01		0.00		892203.43		0.01		1.52		221450.17			0.00		0.72	
Note: ***; p<0.001, **; p<0.01, *; p<0.05																			

For Model BT, the percentage of highly rated *ryokan* and hotels has a positive effect at the 1% significance level for all models: Model BT-O, Model BT-G (G), and Model BT-G (P). The percentage of inbound guests also has a positive effect at the 1% significance level in Model BT-O, Model BT-G (G), and Model BT-G (P). The number of cultural resources has a positive effect in Model BT-O at the 1% significance level, whereas there is no effect in Model BT-G (G) and Model BT-G (P). In terms of travel time, the shortest travel time from Tokyo and shortest travel time from a city with over a million people have a negative effect in Model BT-O at the 5% significance level. However, the result for Model BT-G (G) shows positive and negative effects at the 5% significance level for shortest travel time from Shin-Osaka and the shortest travel time from a city with over a million people, respectively, and the result for Model BT-G (P) indicates positive effects at the 5% significance level for the shortest travel time from Shin-Osaka. Therefore, there was no consistent effect on travel time. The dummy variable of the National Health Onsen Resorts has a negative effect at the 1% significance level in Model BT-G (G) and Model BT-G (P).

As for the results of Model OG, the percentages of highly rated *ryokan* and hotels are significant at the 5% significance level in all models [Model OG-O, Model OG-G (G), and Model OG-G (P)]. The rates of inbound overnight guests have a positive impact on the number of overnight guests at the 0.1% level of significance in all models. As is the case with Model BT, the number of cultural resources has a positive effect at the 5% significance level in the OLS model (Model OG-O), but has no effect in the other models, i.e., Model OG-G (G) or Model OG-G (P). The shortest travel time from Tokyo has a negative effect at the 1% significance level in Model OG-O, but has no significant effect in the other models. Similar to Model BT, the dummy variables of the national recreation hot spring in the GLMMs (OG-G (G) and Model OG-G (P)) have negative effects at the 1% significance level, whereas the same dummy variable has no significant effect in the OLS model (Model OG-O).

In Model FG, in which the dependent variable is the number of inbound guests, the percentage of highly rated *ryokan* and hotels had a positive effect at the 5% significance level in all three models of Model FG. The dummy variable of the National Health Onsen Resorts, just as in Model BT and Model OG, has a significant effect at the 5% significance level in the models assuming GLMM (Model FG-G (G) and Model FG-G (P)), whereas the same dummy variable has no significant effect in the OLS model (Model OG-O)). The number of non-Japanese languages on a tourism association website has a positive impact at the 5% level of significance in all models of Model FG, whereas the number of duty-free stores has no significant impact on the models.

## Discussion

From the AIC, the models of GLMM (gamma) with random effects fit the data better than the OLS models for all dependent variable cases. This suggests that the model fit is better when the unique characteristics of hot spring resorts, which are not captured by independent variables in the OLS model but are captured by random effects in the GLMMs in the present study, are considered. Although some famous hot spring resorts with a long history in Japan, such as Hakone and Atami, may be affected by brand power, other factors, such as the quality of accommodations, the number of tourism resources, and accessibility, are explicitly examined in the OLS model of the present study. In fact, Kamata and Misui (2015) showed that uniqueness is the most significant factor in push motivation for hot springs in Japan [24]. Therefore, when considering the demand for hot spring resorts, which seems to be affected by unique attractiveness, which is unlikely to show up in the usual public statistical figures, the GLMM including random effects may be more suitable for considering the impact on demand, rather than the simple OLS model. The RMSEs in the GLMM models are smaller than those in the OLS models. The RMSEs in the GLMM models in Model BT-G (G) and Model BT-G (P), whereas gamma Models (OG-G (G) and FG-G (G)) have slightly lower RMSEs than Poisson Models (OG-G (P) and FG-G (P)), respectively. This is natural because the error distribution in Model OG-G (G) and Model FG-G (G) assumes a continuous function. However, since these dependent variables are non-negative count data, it seems more reasonable to assume a Poisson error distribution.

The GLMM estimation results suggest that 5) the quality of accommodations and the proportion of inbound 3) overnight guests contribute to the overall demand for famous hot spring resorts in Japan, i.e., the number of overnight guests and 2) the amount of bath tax revenue. It has been reported that the quality of accommodations is an important factor for customer satisfaction in hot springs in Taiwan and China [17, 19, 20]. It is natural to assume that a high level of customer satisfaction has a positive impact on demand, and it is reasonable that the quality of accommodations also has a positive impact on demand for hot springs in the present study. This result suggests that the quality of accommodations, which influences customer satisfaction in health resorts and medical spas in Taiwan and China, is a factor that directly affects the demand for hot springs in Japan. The significant positive parameter results for the proportion of inbound overnight guests indicate that the success in obtaining inbound guests to a hot spring resort among a guest composition is significantly related to the total demand for the hot spring. This may mean that hot spring resorts with large accommodation demand are more successful in attracting inbound guests due to large-scale marketing activities and the brand power associated with the large scale of these resorts. In this case, the proportion of inbound overnight guests may not be a cause of accommodation demand. On the other hand, when closely observing each hot spring individually, there are some hot springs that rank high in terms of the number of overnight guests and low in terms of the number of inbound overnight guests, even though the Pearson correlation coefficient between the overall number of overnight guests and the overall number of inbound guests is relatively high at approximately 0.78. For example, Kinugawa/Nikko Yumoto, which are ranked 4th in terms of the number of overnight guests, are ranked 22nd in terms of the number of inbound overnight guests. This may be related to the difference in the impact of the brand and the marketing strategy of the hot spring on domestic and inbound demand. Although the number of inbound tourists to Japan increased 3.7 times from 2010 to 2019 [37], domestic overnight demand in Japan is still more dominant than inbound overnight demand. However, since the volume of domestic guest demand was stable throughout the 2000s before the COVID-19 pandemic, the percentage of inbound overnight guests may imply the degree of inbound customer acquisition of a hot spring before the COVID-19 pandemic. Therefore, the percentage of inbound overnight guests may be a useful indicator associated with demand at Japanese hot springs in the relevant period.

While it has been suggested that 3) the number of inbound overnight guests is related to 12) the number of non-Japanese languages on a tourism association website, there is no significant effect of 13) the number of duty-free stores. Hsu et al. (2009) reported that the web function has a significant effect on loyalty in a survey of hot springs in Taiwan [11]. Thus, if we assume that hot springs with high loyalty have high demand for their accommodations, then it is not surprising that web functions for inbound markets, such as the language of the homepage, have a significant effect on the demand. In addition, it has been pointed out that the language barrier hinders inbound demand for hot springs in Japan [1, 2, 5]. This result suggests that the enhancement of supported languages on websites, especially for Japanese hot springs, may be effective in increasing inbound demand. Shopping is one of the top expectations of inbound visitors to Japan [9], and, in fact, the duty-free system for inbound tourists is currently being enhanced by tax reform in Japan [38]. Although this enhancement is aimed at increasing the amount of consumption by inbound tourists and not directly at increasing inbound demand, at least the results of the present study suggest that establishing duty-free stores in hot springs does not contribute to increasing the inbound overnight demand at hot springs. In addition, 5) the percentage of high-rated *ryokan* and hotels, which was significant for 2) the bath tax or 1) the number of overnight guests, was also significant for 3) the number of inbound guests.

Although 6), 7) tourism resources and the surrounding environment have been shown to be important to customer service at hot springs [11, 17, 19, 21], the results of the present study show that no significant results were found for the GLMMs, whereas significant effects were identified in the OLS model. Hence, consistent results were not obtained in our analysis.

Kamata et al. (2010) showed that 8), 9), 10) accessibility from Tokyo and Osaka influences the attractiveness of hot springs [8]. However, in the present study, although some significant effects are found in Model BT, the results are not consistent across all models. Furthermore, Model BT-G(G) had an unreasonable positive effect on 9) the shortest travel time from Shin-Osaka. These results cannot positively support the results obtained by Kamata et al. (2010) [8]. However, they used the NITAS data for accessibility data, which considers the time and cost of railways and other modes of transport [39]. However, the present study only considers railways. It is possible that the effects of travel time by airplane and car could not be fully captured by our data. In addition, the number of hot springs in Japan analyzed by Kamata et al. (2010) was 20 [8], whereas the present study analyzed more than four times that number. In the data used in the present study, there is a large difference in the number of overnight guests and the amount of bath tax revenue among different hot springs located at similar distances from 10) urban areas (large bullet train stations). Therefore, the data used in the present study cover a wider variety of hot springs than the data treated in the previous study, which may have prevented consistent accessibility effects from being estimated.

As for 11) the National Health Onsen Resorts, the criteria for potential resorts to be certified includes not only the quality of water at the hot springs, but also the safety of the hot spring site, such as the placement and availability of doctors, the assignment of personnel to instruct ways of bathing, and efforts related to hot spring sanitation and disaster prevention. It has been pointed out that the safety of hot spring resorts and the quality of human resources are important factors for the competitiveness of hot spring resorts [7, 20, 21]. The present study shows that there is no significant effect in the OLS models and a significant negative effect in all GLMMs. This means that the demand for a hot spring certified as one of the National Health Onsen Resorts is lower than that for a hot spring that is not certified. In order to be certified, it is necessary that the source of spring water be appropriate for a sanatorium spring, and the source is evaluated from the perspective of medical treatment and recreation. Therefore, the hot spring is different from the perspective of increasing demand, such as the attraction for recreational purposes with family and friends or attracting new inbound demand. The present study suggests that there might be a trade-off between emphasizing efforts to increase demand and emphasizing recuperation and recreation.

Since customer service and loyalty have already been treated as important factors in hot springs, it is natural that review scores in a Social Networking Service (SNS) are shown as significant indicators of demand for hot springs. Furthermore, considering that domestic overnight demand was stable prior to the COVID-19 pandemic, such a finding is reasonable in Japan, where inbound demand continued to grow until the COVID-19 pandemic, increasing the ratio of inbound demand leads to an increase in total overnight demand in a hot spring during the same period. On the other hand, the factors of accessibility and the environment of the hot springs do not have significant effects in our regression models with various dependent variables. These factors have been analyzed as important in tourism studies on hot spring resorts, but these factors have been estimated for a limited number of hot spring site samples. Our results suggest that these factors may not be robust when demand is evaluated based on the actual demand data for a relatively large number of hot springs.

Note that the factors that are estimated to significantly affect demand across all regression models, i.e., the quality of accommodations in a hot spring and the degree of dependency for inbound demand, can be changed relatively easily by local efforts. This is not true for the quality of the hot spring water and the number of tourism resources located in the hot spring resort. In order to increase the overnight demand, which is directly related to the revenue of a hot spring resort, it is important to improve the quality of accommodations in a hot spring resort, i.e., the quality of customer service, and to refine marketing strategy to increase the percentage of demand from non-domestic markets. This suggestion is based on analyses of data from more than 80 Japanese hot spring resorts. Although this suggestion seems natural and general, this may be a common policy for many hot spring resorts to increase demand. However, it is unclear whether these suggestions will be valid after the pandemic. Shin et al. (2022) suggested that trust for destination is a significant factor in future intention to travel abroad after the pandemic [29]. Thus, including information that builds trust for inbound guests in Web sites of hot spring resorts might also be efficient after the pandemic.

## Conclusion

In the present study, we estimated various contributing factors that have been considered to be important in previous hot springs studies by OLS and GLMM regression analysis. This analysis uses public statistic and Web data for mainly 2018, which was before the COVID-19 pandemic. The results suggest that the quality of accommodations and the degree of dependency of inbound demand have robust effects on the overnight demand for hot springs.

The number of hot springs analyzed in the present study is over 80, which is larger than that in previous hot springs studies, and major Japanese hot springs throughout the country are included in the analysis. Unlike most previous hot springs studies, which analyzed the impact on customer service, loyalty, and customer satisfaction based on questionnaire surveys, the present study focused on the factors that affect the actual number of overnight guests and the actual number of users of hot springs. Even if it is assumed that customer service, loyalty, and customer satisfaction have a positive impact on actual demand, the impact does not seem to be fully transmitted to the actual demand. Therefore, the present study may examine the impact with more stringent criteria than existing hot springs studies. Limitations of the present study are that we do not consider the quality of cuisine, which has been considered to be important in previous studies, and that only railways are considered in terms of accessibility. In addition, since data from multiple years were not analyzed, it has not been verified whether the results of the present study are equivalent across multiple years. Therefore, the addition of more factors and the expansion of the analysis years are considered as future tasks.

The present study does not highlight new factors that have not been considered to be important in previous tourism research on hot spring resorts. However, the results of the present study may provide priorities for policies to improve demand across a wide range of hot springs. In addition, the impact of the pandemic has not completely disappeared and is still ongoing as of the end of June 2022 in Japan. Therefore, we could not yet reach an overall conclusion as to the impact of the pandemic on hot spring resorts. The results of the present study, which covers the period before 2020 when data for domestic and inbound tourist are available without any pandemic influence, provide an objective baseline for investigation of the transitional change of demand factors based on broad public data through the pandemic. Thus, it is expected that our findings with robust regression methods will improve a reference point for comparative studies when the pandemic situation is stabilized.

## Supporting information

S1 File Data_availability_statement. (DOCX)

S2 File Repository_Japanese_hot_springs_data. (CSV)
