# Supplementary material for: Factors contributing to tourism demand at major Japanese hot springs
Source: PLoS One. 2022 Sep 15;17(9):e0274681. doi: 10.1371/journal.pone.0274681 (PMC9477256; doi:10.1371/journal.pone.0274681)
Supplement: S1 File — (DOCX) [file pone.0274681.s001.docx]

Data Availability Statement

All of the data in the present study were collected and analyzed in accordance with the terms and conditions of each data source for the 13 variables [1) to 13)] indicated in Table 2 in the manuscript. Collection of all data requires no special technology. Most of the data are publicly and freely available from the indicated sources. The data are listed as List A indicated below [numbers, such as 2), are the numbers assigned to variables indicated in Table 2]:

**List A: List of Variables and their Publicly Accessible Data Sources**

- 2) Bath taxes (JPY) from local financial situation survey (Ministry of Public Management, Home Affairs, Posts and Telecommunications) https://www.soumu.go.jp/english/index.html
- 4) Ryokan and Hotels from Economic Census Survey for Business Activity (Ministry of Internal Affairs and Communications) https://www.stat.go.jp/english/data/e-census.html
- 6) Natural resources from Tourism Resources Directory (Japan Travel Bureau Foundation) https://www.jtb.or.jp/page-search-tourism-resource/ (in Japanese)
- 7) Cultural resources from Tourism Resources Directory (Japan Travel Bureau Foundation) https://www.jtb.or.jp/page-search-tourism-resource/ (in Japanese)
- 11) Dummy variable of the National Health Onsen Resorts from List of the National Health Onsen Resorts (Ministry of the Environment of Japan) https://www.env.go.jp/nature/onsen/area/ (in Japanese)

However, making use of some of the data sources indicated in Table 2 requires payment, as indicated in the terms and conditions of the data source. So, such data, which cannot be accessed freely, are listed along with their source in List B:

**List B: List of Variables and their Proprietary Data Source**

- 1) Overnight guests from Japan Voyage Navigator, https://kankouyohou.com/en/
- 3) Inbound guests from Japan Voyage Navigator, https://kankouyohou.com/en/
- 13) Duty-free stores from Japan Voyage Navigator, https://kankouyohou.com/en/

In addition, some variables are restricted according to collection time. In other words, data were collected without the possibility of tracing the data back to a different year. Such variables and their data sources are given as List C:

**List C: List of Variables for Data with Specific Dates Indicated in Table 2 and their Data Sources**

- 5) Percentage of highly rated Ryokan and hotels from Tripadvisor, https://www.tripadvisor.jp/
- 8) Shortest time from Tokyo Station (minutes) from Route Information (Yahoo!Japan), https://transit.yahoo.co.jp/ (in Japanese)
- 9) Shortest time from Shin-Osaka Station (minutes) from Route Information (Yahoo!Japan), https://transit.yahoo.co.jp/ (in Japanese)
- 10) Shortest time from a station in a big city where the Shinkansen stops (minutes), from Route Information (Yahoo!Japan), https://transit.yahoo.co.jp/ (in Japanese)
- 12) Non-Japanese languages supported by the tourism association from 85 websites of the tourism association of each municipality (all are publicly accessible, directly or through such web sites as https://www.homemate-research.com/tour_search/ )

The data in List C cannot be traced back to the data for the year in Table 2 in the manuscript because of the setting of the data source. Therefore, in order to maintain the necessary level of data sharing and replicability, we set up a **minimum data set** in the Supporting information file as “S2_File. Repository_Japanese_hot_springs_data.csv.” We stored in the file the publicly available data in List A and the actual data we used for variables in List C, whereas only the proprietary data in List B are not included.

Anyone who obtains access to the restricted data source in List B (Japan Voyage Navigator) according to the terms indicated on the website can add data to our **minimum data set** in the Supporting information file (S2 File. Repository_Japanese_hot_springs_data.csv) and can completely reconstruct our data set, consisting of all data in Lists A, B, and C.

All relevant information regarding data access is in the Supporting Information files, namely this document, S1 File. Data_Availability_Statement.docx.

In the **minimum data set** in the Supporting information file (S2_File. Repository_Japanese_hot_springs_data.csv), each item below corresponds to a variable in Table 2:

*destination*: 85 municipalities in the manuscript

*tax*: 2) Bath taxes (JPY)

*ryokan.hotels*: *_*4) Ryokan and Hotels

*quality*: 5) Percentage of highly rated ryokan and hotels

*nat*: 6) Natural resources

*cul*: 7) Cultural resources

*time_tokyo*: 8) Shortest time from Tokyo Station (minutes)

*time_osaka*: 9) Shortest time from Shin-Osaka Station (minutes)

*time_city*: 10) Shortest time from a station in a big city where the Shinkansen stops (minutes)

*reg_dummy*: 11) Dummy variable of the National Health Onsen Resorts

*lang*: 12) Non-Japanese languages supported by the tourism association
